# Using Wearable Sensors to Assess Freezing of Gait in the Real World

**DOI:** 10.3390/bioengineering10030289

**Published:** 2023-02-23

**Authors:** David S. May, Lauren E. Tueth, Gammon M. Earhart, Pietro Mazzoni

**Affiliations:** 1 Program in Physical Therapy, Washington University School of Medicine, St. Louis, MO 63108, USA; 2 Department of Neurology, Washington University School of Medicine, St. Louis, MO 63110, USA; 3 Department of Neuroscience, Washington University School of Medicine, St. Louis, MO 63110, USA

**Keywords:** Parkinson’s disease, freezing of gait, gait, wearable sensors, home environment, sensors for rehabilitation

## Abstract

Freezing of gait (FOG) is a debilitating symptom of Parkinson’s disease (PD) that remains difficult to assess. Wearable movement sensors and associated algorithms can be used to quantify FOG in laboratory settings, but the utility of such methods for real world use is unclear. We aimed to determine the suitability of our wearable sensor-based FOG assessment method for real world use by assessing its performance during in-clinic simulated real world activities. Accuracy of the sensor-based method during simulated real-world tasks was calculated using expert rated video as the gold standard. To determine feasibility for unsupervised home use, we also determined correlations between the percent of active time spent freezing (%ATSF) during unsupervised home use and in-clinic activities. Nineteen people with PD and FOG participated in this study. Results from our sensor-based method demonstrated an accuracy above 90% compared to gold-standard expert review during simulated real-world tasks. Additionally, %ATSF from our sensor-based method during unsupervised home use correlated strongly with %ATSF from our sensor-based method during in-clinic simulated real-world activities (ρ = 0.73). Accuracy values and correlation patterns suggest our method may be useful for FOG assessment in the real world.

## 1. Introduction

It is estimated that over 80% of people with Parkinson’s disease (PD) eventually experience a phenomenon called freezing of gait (FOG), which is a “brief episodic absence or marked reduction of forward progression of the feet despite the intention to walk [1,2].” FOG causes falls and is often resistant to levodopa [3,4], the most commonly prescribed medication to treat the symptoms of PD. FOG is episodic in nature [5] and is difficult to reliably elicit and assess. FOG can be directly observed in a clinical or laboratory setting and can be quantified through video review by a movement disorders expert. However, FOG often disappears when people with FOG consciously focus on walking, such as when being observed by a clinician [6,7,8]. Factors that trigger FOG, such as anxiety [9,10], are difficult to safely reproduce while walking in a clinic setting. Many clinic settings and hallways are broad and free of obstacles, whereas FOG often occurs when navigating narrow spaces and turning [6]. As such, FOG severity, as observed in a clinical or laboratory setting, is unlikely to reflect FOG severity in the real world. Video review is also onerous, time-consuming, and not practical in a clinical setting. There is therefore a need for an adequate approach to assess FOG severity.

Clinicians and researchers currently rely primarily on the New Freezing of Gait Questionnaire (NFOG-Q) to assess FOG severity. The NFOG-Q is a useful screening tool to differentiate between freezers and non-freezers and shows a high reliability of scores between people with PD and their caregivers [11,12]. However, as a questionnaire, it is inherently subject to the problem of recall bias. It does not correlate with observed FOG severity in lab settings [13], and NFOG-Q has only modest test–retest reliability and is not responsive to small changes in FOG [14]. New methods are needed to assess FOG, as clinical trials and clinicians rely on accurate and responsive outcome measures to assess potential responses to treatment.

In recent years, wearable inertial measurement units (wearable sensors) and associated algorithms have been proposed as methods for assessing real-world FOG severity. Researchers in our laboratory have worked with engineering colleagues to develop one such method [15,16]. Our method uses a two-stage detector for determining the zero-velocity and trembling events in gait and then utilizes a point-process filter to calculate the probability of FOG during these events. Our method was validated in a laboratory setting, can be adapted for individual gait patterns, and can automatically detect FOG in real time [15,16,17]. However, this algorithm has not yet been tested outside of structured gait and turning tasks.

It is important to determine whether wearable sensor methods such as ours can be used to detect FOG during real-world activities outside the laboratory or clinic, as some people with FOG do not reliably demonstrate FOG in a laboratory or clinical setting. Wearable sensors could theoretically be used in the future as the “gold standard” method for assessing FOG severity. While several sensor-based methods for assessing FOG have been developed, it is unclear whether such methods are capable of accurately assessing FOG during unstructured spontaneous movements of daily life. In one recent study, a sensor-based FOG assessment method was shown to detect significantly more presumed FOG events during unsupervised home use in people with known FOG compared to people without known FOG [18]. Little else is yet known about the performance of sensor-based FOG assessment methods in the real world. An important first step is to test the sensors while performing activities designed to mimic instrumental activities of daily life (IADLs) in a simulated home setting within the laboratory and compare the performance of our sensor-based measures to the gold-standard video rating method. Additionally, it is important to explore how our sensor-based methods in lab compare to unsupervised home use. These methods would allow us to determine whether our sensor-based FOG assessment method could be useful in a home environment.

The primary objective of this study was to determine the accuracy of our sensor-based FOG assessment method while performing tasks designed to mimic IADLs in a simulated home setting within the laboratory. As a secondary outcome, we compared the percent of active time spent freezing (%ATSF) according to our sensor-based method to that of expert video review during (1) laboratory-based gait tasks designed to maximally elicit FOG, (2) tasks designed to mimic IADLs in a simulated home setting within the laboratory, and (3) unsupervised home use. Using the sensors in these three manners allowed us to also explore correlation patterns which may elucidate the utility of our approach for unsupervised use in the real world.

## 2. Materials and Methods

### 2.1. Participants

Participants were recruited from the Movement Disorders Clinic at Washington University in St. Louis. For inclusion in this study, all participants were required to: (1) be age 30 or older; (2) have a diagnosis of “clinically definite PD”’; (3) have a history of FOG in the past 2 months according to the treating movement disorders neurologist; (4) be able to walk for at least 100 feet with or without an assistive device. Participants were excluded from the study if they had: (1) any pre-existing medical conditions that would inhibit full participation in the study tasks or (2) cognitive impairments indicated by a Mini-Mental Status Exam (MMSE) score of <24 [19]. Written informed consent was obtained from each participant in accordance with the Declaration of Helsinki and the policies and procedures of the Human Research Protection Office at Washington University in St. Louis. This study was approved by the Institutional Review Board at Washington University in St. Louis (protocol 202004254).

We recruited and tested 20 participants. One participant’s data were excluded from analysis because she was unable to complete the gait tasks (described below) without prolonged pauses for rests in the middle of the tasks. The research team determined at the time of testing that this participant’s data would be excluded because the pauses would disrupt the human expert’s assessment of FOG.

### 2.2. Procedure

All participants arrived at Washington University for one in-person visit. Participants were instructed to take their regular doses of levodopa at their usual times on the day of the in-person visit. Disease severity was measured at the in-person visit with the Movement Disorders Society Unified Parkinson’s Disease Rating Scale (MDS-UPDRS) [20]. The MDS-UPDRS Part III was administered by a certified examiner. At the beginning of the in-person visit, each participant also completed the NFOG-Q.

At the in-person visit, participants completed a series of walking and turning tasks designed to elicit FOG in a clinical setting (clinic FOG tasks) and a series of tasks in a simulated apartment room designed to mimic activities that participants might perform in the home and community in daily life (simulated IADL tasks), while wearing inertial measurement units (sensors) to detect FOG. All clinic FOG tasks and simulated IADL tasks were video recorded for review. After the in-person visit, participants took the sensors home and wore them during their daily activities for three days (home portion) before returning them to the study team. No video recording occurred during the home portion of the study. Three sensors were worn by participants throughout all components of the study, with one sensor placed on the dorsum of each foot (in pouches strapped onto the participant’s shoes) and one sensor placed over the left hip (in a pouch clipped to a belt). Participants completed three clinic FOG tasks and four simulated IADL tasks during the in-person visit. Instructions for each clinic FOG task and each simulated IADL task were highly standardized to avoid differences in instruction between participants. The same two research team members tested all participants, with one always giving instructions and the other never speaking during the tasks and simply walking behind the participant at all times for safety. The highly disciplined protocol was intended to balance the number of left and right turns in each task and to avoid positive or negative emotional cues during testing, as it was clear during pilot testing that these factors influenced the probability of FOG occurrence. Detailed information about each of these tasks is provided in Appendix A.

The three clinic FOG tasks were designed to incorporate multiple triggers of FOG drawn from previous studies [13,21] and our own pilot testing. The clinic FOG tasks were labeled: (1) Hallway Pivot, (2) Go Outside and Turn (GOT), and (3) 360-Degree Turn. The Hallway Pivot task consisted of walking in a straight line down a hallway, turning 180 degrees toward the left or right when instructed, and then continuing to walk in the opposite direction. The GOT task consisted of walking out of a clinical examination room through an open door into a hallway, making a 180 degree turn within an open box marked on the floor in the middle of the hallway, and then walking around the perimeter of the box before walking back into the clinical examination room. The 360 Degrees Turn task was performed by standing in place and turning 360 degrees when instructed. Each clinic FOG task was performed as a single task and in a dual-task format with a concurrent cognitive task (counting down in steps of three, starting from a 3-digit number randomly chosen for each trial). Each participant completed each clinic FOG task at least once per direction (i.e., turning once toward the right and once toward the left) as a single task and once per direction as a dual task.

The simulated IADL tasks consisted of (1) vacuuming the floors of the simulated apartment room (vacuum task), (2) emptying a dishwasher and putting dishes away in clearly marked cabinets and drawers positioned around the simulated apartment room (dish task), (3) sitting in a recliner with a footstool for at least ten minutes (sitting task), and (4) walking through the hallways, doorways, and elevators of the clinic building to a given destination to simulate navigating a public building such as a clinician’s office or store (community task). Each participant completed each simulated IADL task once.

After the in-person visit, participants wore the sensors at home for three days. Participants were instructed to remove the sensors from the pouches each night for charging while sleeping and were instructed not to remove the pouches from the shoes until the study was complete. Participants were asked to wear these shoes with the sensors donned as much as possible while awake but were given permission to temporarily remove these shoes if desired for aesthetic purposes (e.g., while wearing more formal clothing for dinner at a restaurant) or to protect the devices (e.g., bathing). Each participant and any individuals present who resided with the participant were educated in how to charge the sensors each night and how to set them in the correct positions the next morning after charging. Home instructions are detailed in Appendix B. The sensors were color-coded to reflect their assigned location (right foot, left foot, left hip). This coding was intended to facilitate the nightly task of removing the sensors from their pouches at bedtime and placing them back in the correct pouch the next morning. There was no difference in the technical specifications of sensors of different colors.

### 2.3. Equipment

Foot and left hip motion were recorded by inertial measurement units (IMUs) that included tri-axial gyroscopes and accelerometers housed in wearable sensors. These consisted of Physilog 6 sensors (Gait up SA, Lausanne, Switzerland) sampled at 128 Hz for the first 6 participants and ActiGraph GT9X Link (ActiGraph, Pensacola, Florida) sampled at 100 Hz for the remaining participants. The Physilog P6 sensors contain 3D accelerometers (range: ±16 g) and 3D gyroscopes (range: ±2000 deg/s) with sampling capability up to 512 Hz and max storage of 450 MB. Their battery capacity at the sampling frequency used in our study (128 Hz) was ~20 h. The Actigraph GT9X Link sensors contain 3D accelerometers (range: ±16 g) and 3D gyroscopes (range: ±2000 deg/s) with a sampling rate of 100 Hz and a storage capacity of 4 GB. The battery capacity at the sampling frequency of 100 Hz was ~19.5 h. Participants wore the sensors starting after getting dressed in the morning and set them to charge before bedtime on each of the 3 evenings of the home session. Data from Physilog sensors were down-sampled from 128 to 100 Hz to match the sampling rate of the Actigraph sensors, used by the remaining 13 participants, using a Hanning-windowed sinc filter (Igor version 8.04; WaveMetrics, Lake Oswego, Oregon). All sensor data were analyzed off-line by first downloading sensor data in proprietary binary format and then converting to plain text tables. The time required to pre-process sensor data in this manner was similar (~15 min) for the two sensor brands. The change in sensors was necessitated by breakage of one sensor that could not be replaced due to pandemic-related semiconductor chip shortages. Data were downloaded for offline analysis after the in-person visit and again after home use.

### 2.4. Data Analysis

**Figure 1:**
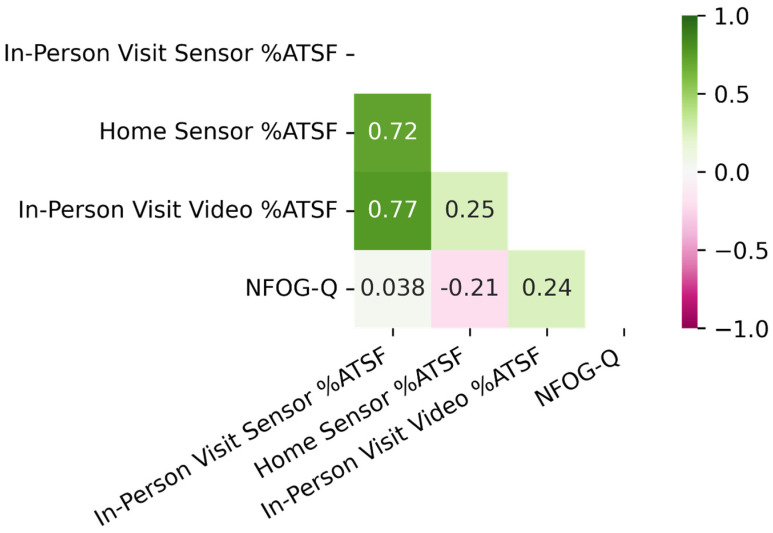
In-Person Visit Correlations. *n* = 19. Values represent Spearman’s Rho values. “In-Person Visit” refers to Clinic FOG Tasks + Simulated IADL tasks. “Sensor %ATSF” refers to sensor-based percent active time spent freezing, and “Video %ATSF” refers to video-based percent of active time spent freezing, as determined by an expert rater upon video review.

**Figure 2:**
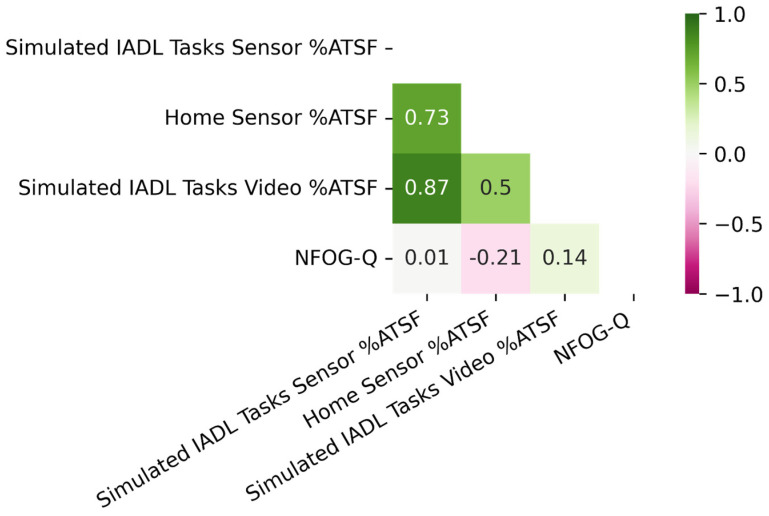
Simulated IADL Task Correlations. *n* = 19. Values represent Spearman’s Rho values. Simulated IADL tasks refer to the Vacuum, Dish, Sitting, and Community tasks, which all occurred during the in-person visit. “Sensor %ATSF” refers to sensor-based percent of active time spent freezing, and “Video %ATSF” refers to video-based percent of active time spent freezing, as determined by an expert rater upon video review.

For consistency across participants, data collected by the Physilog sensors were downsampled to 100 Hz before analysis to match the sampling rate of the ActiGraph sensors. We detected FOG events in sensor data by first calculating probability of FOG using the Probability of Freezing of Gait (pFOG) algorithm developed by Prateek et al. [15]. The estimation process to detect FOG was carried out through a set of computations that included detection, navigation, and filtering modules, illustrated in Figure 1 of Ref. [15]. FOG events were the times when pFOG for either foot was above a threshold of 0.7. The Python programming language [22] was used for cleaning, processing, and analysis of data. The pandas [23], NumPy [24], SciPy [25], and tkinter [26] packages were used throughout various steps of this work. Matplotlib [27] and Seaborn [28] were used to generate Figure 1 and Figure 2. Events separated by less than 2 s were merged into single FOG events.

The choice of the pFOG algorithm was based on its detection performance in previous testing when compared to multiple other detection algorithms for FOG [15]. This algorithm has so far been applied to the present project’s dataset and to datasets described in [15]. The analysis was carried out with custom software that implements the pFOG algorithm in Python (version 3.6) on a 2016 Macbook Pro laptop running MacOS version 10.13 and Darwin kernel version 20. The data processing Python scripts were called by a Jupyter Notebook version 4.1. The time for the estimation process was about 90 min for one lab session and about 9 h for a 3-day home session.

Foot sensor data were analyzed only for segments when the participant was non-sedentary, as indicated by hip sensor data. We considered sedentary all time segments with total hip angular velocity (sum of absolute value of hip angular velocity in each of the three axes) less than 1 deg/s and excluded these from analysis [18]. An example is a participant sitting in a chair and bouncing his or her leg up and down. Non-sedentary (active) times were calculated as times when total hip angular velocity (filtered through a centered 20 s moving average window) in any plane was above 1 deg/s. For all portions of the study, including the in-person visit and the home portion of the study, sedentary times were removed from analysis.

For the in-person visit, only data from during the clinic FOG tasks and the simulated IADL tasks were analyzed. Video review was considered the “gold standard” for detecting FOG during the in-person visit. A licensed physical therapist with experience in assessment and treatment of people with PD analyzed the video recordings to determine when FOG occurred during the clinic FOG tasks and the simulated IADL tasks. In accordance with past work [18], the beginning of a FOG episode was defined as the moment when the gait pattern appeared arrested or it appeared as if the participant was trying unsuccessfully to initiate or continue locomotion or turning. The end of a FOG episode was defined as the moment when an effective step had been performed and was followed by at least one more effective step.

Any times for which the sensors and video review both detected FOG were labeled as true positives, and any times for which neither method detected FOG were labeled as true negatives. Accuracy was determined by calculating the ratio of the true positives plus true negatives to the total length (in samples) of the dataset. Accuracy was calculated for the whole in-person visit (clinic FOG tasks and simulated IADL tasks) as well as for each simulated IADL task individually to determine if certain IADL tasks were associated with greater or lesser detection accuracy.

In order to explore the amount of time spent freezing, the rater summed the total amount of time spent freezing for each participant and the total amount of time spent performing each task. The remaining data were used to calculate the video-based percent of active time spent freezing (Video-Based %ATSF) for the whole in-person visit (clinic FOG tasks plus simulated IADL tasks) and separately for the simulated IADL tasks only.

For both the in-person visit and the home portion of the study, the sensor-based percent of active time spent freezing (Sensor-Based %ATSF) was calculated by dividing the total amount of time spent freezing by the total amount of active time and multiplying the result by 100. For the in-person visit, Sensor-Based %ATSF was calculated for the whole visit (clinic FOG tasks plus simulated IADL tasks) and separately for the simulated IADL tasks only.

Without video recording in the home, it is impossible to calculate the accuracy of our sensor-based FOG detection method during home use. Therefore, we examined the correlations between Video-Based %ATSF measures from the in-person visit and Sensor-Based %ATSF at home to determine whether the amount of FOG detected for each person during the in-person visit correlates with the amount of FOG detected for each person at home. We also examined the correlations between Video-Based %ATSF measures and the Sensor-Based %ATSF measures from the in-person visit to provide additional information about performance of the sensor-based FOG detection during clinic FOG tasks and simulated IADL tasks. Additionally, we examined the correlation between the NFOG-Q and all %ATSF measures to determine how the NFOG-Q relates to observed measures of FOG severity. All %ATSF scores, as well as NFOG-Q scores, were checked for normality using Shapiro–Wilk tests. Because each set of %ATSF scores was found to have a non-normal distribution, Spearman’s rank correlations (Spearman’s ρ) were used throughout.

## 3. Results

Of the twenty participants who initiated data collection, 19 completed the study and were included in the present analysis. Participant demographics are provided in Table 1.

Out of 19 participants, two did not have any observed FOG during the in-person lab visit. Five participants did not have any observed FOG during the simulated IADL tasks. All participants with observed FOG during the simulated IADL tasks also had observed FOG during the clinic FOG tasks.

The primary objective of this study was to determine the accuracy of our sensor-based FOG assessment method while performing tasks designed to mimic IADLs in a simulated home setting within the laboratory. The mean accuracy across participants (*n* = 19) for each simulated IADL task is in Table 2. The mean accuracy across participants (*n* = 19) for the entire in-person visit, i.e., clinic FOG tasks plus simulated IADL tasks, is also shown in Table 2. The mean accuracy for each simulated IADL task, as well as for the entire in-person visit, was above 90%.

Following Mancini et al., we included a third sensor at the waist to filter out sedentary time from analysis. Our method succeeded in excluding only the times which were very likely to be sedentary. During the in-person visit, this modification only made a difference for two out of 19 participants, and it only filtered out time during the sitting task for both participants. The addition of this modification improved accuracy during the sitting task for these two participants. No times during other tasks were filtered out as sedentary for any participants.

Across participants with observed FOG during the lab visit, the average duration of FOG episodes was 5 s for the entire in-person visit. For the 14 participants with observed FOG during the simulated IADL portion of the in-person visit, the average duration of FOG episodes was also 5 s. Overall, clinic FOG tasks elicited more freezing than the simulated IADL tasks.

Secondary analyses explored the %ATSF. The mean %ATSF across all 19 participants is shown in Table 3 for each sensor and video-based measure.

Correlations between the NFOG-Q, measures from the entirety of the in-person visit, and Sensor-Based %ATSF from the home (*n* = 19) are shown in Figure 1. The Sensor-Based %ATSF from the in-person visit correlated strongly with the Video-Based %ATSF from the in-person visit (ρ = 0.77). The Sensor-Based %ATSF from the home also correlated strongly with the Sensor-Based %ATSF from the in-person visit (ρ = 0.72). The Sensor-Based %ATSF from the home showed a weak correlation with the Video-Based %ATSF from the in-person visit (ρ = 0.25). The NFOG-Q showed no strong correlation with any objective measures of %ATSF from the entirety of the in-person visit or from the home.

Correlations between the NFOG-Q, measures from the simulated IADL task portion of the in-person visit, and Sensor-Based %ATSF from the home (*n* = 19) are shown in Figure 2. The Sensor-Based %ATSF from the simulated IADL tasks and the Video-Based %ATSF from the simulated IADL tasks correlated especially strongly (ρ = 0.87). The Sensor-Based %ATSF from the simulated IADL tasks also correlated strongly with the Sensor-Based %ATSF from the home (ρ= 0.73). The Sensor-Based %ATSF from the home showed a moderate correlation with the Video-Based %ATSF from the simulated IADL tasks (ρ = 0.50). The NFOG-Q showed little to no correlation with the Video-Based %ATSF from the simulated IADL tasks or the Sensor-Based %ATSF from the simulated IADL tasks.

## 4. Discussion

We examined the performance of our sensor-based FOG assessment during structured laboratory gait tasks, simulated real world activities, and three days of unsupervised home use. Our sensor-based method was successfully utilized in the home by 19 participants. High accuracy values during simulated real-world activities, correlations between our sensor-based approach and expert video review, and correlations between home and laboratory measures of FOG suggest that our sensor-based FOG assessment method may be useful for unsupervised use in the real world and is capable of identifying FOG during real world activities.

The simulated IADL tasks introduced in this study required participants to move and walk in ways that our sensor-based FOG assessment method had not yet encountered, such as side-stepping, repeatedly stepping forward and backward (during the vacuum and dish tasks), and sitting in a recliner for at least ten minutes, a task which typically involves occasional movement and adjustment of the lower extremities. Prior to this study, it was unclear how our sensor-based FOG assessment method would perform during such activities. However, the accuracy of our sensor-based method was above 90% across the entire in-person visit and during each of the simulated IADL tasks, demonstrating the ability of our sensor-based method to assess FOG during real world activities.

Interestingly, accuracy values were even higher for the simulated IADL tasks than for the in-person visit as a whole. One potential explanation for this could be that participants spent less time freezing during the simulated IADL tasks compared to the in-person visit as a whole. Even though we merged any FOG episodes detected by our sensor-based method that occurred within two seconds of each other, our sensor-based method appears to still be detecting long FOG episodes as shorter, successive bursts of FOG episodes and therefore undercounting the %ATSF relative to the human rater. Indeed, Sensor-Based %ATSF was lower than Video-Based %ATSF for the in-person visit and the simulated IADL tasks, and overall accuracy decreased with increasing Video-Based %ATSF for the in-person visit. While the two second threshold for merging episodes was found to be optimal for the sensor-based FOG assessment method developed by Mancini et al. [18], future work could examine whether this threshold is most optimal for our sensor-based method and whether other approaches could be used to modify the algorithm so that long FOG episodes are more accurately detected. However, the observed accuracy in this study is still quite high, and our approach already appears to be capable of assessing FOG severity in real-world settings for this sample of participants.

The Sensor-Based %ATSF from the in-person visit correlated strongly (ρ = 0.77) with the Video-Based %ATSF from the in-person visit. Therefore, during the in-person visit, participants with greater amounts of FOG according to expert review tended to show greater amounts of FOG according to our sensor-based method. This relationship was even stronger for the simulated IADL task portion of the in-person visit (ρ = 0.87). These results suggest that our sensor-based method is capable of accurately assessing FOG during structured clinic FOG tasks and during simulated IADL tasks.

Correlations between Sensor-Based %ATSF in the home and other measures of FOG severity require more careful interpretation, as we did not have video recordings of participants in their homes and therefore lack a gold standard for comparison. Nonetheless, results suggest our sensor-based method can assess FOG in the home. Sensor-Based %ATSF in the home correlated strongly with Sensor-Based %ATSF from the in-person visit and the simulated IADL task portion of the in-person visit. These relationships suggest our sensor-based FOG assessment method was capable of accurately assessing FOG in the real world, and that the addition of unsupervised, unstructured movement patterns did not significantly confound our detection method. Correlations between Sensor-Based %ATSF in the home and Video-Based %ATSF from the in-person visit were not as strong, though the correlation was stronger for the simulated IADL task portion of the visit (0.50) than for the in-person visit as a whole (0.25). It is, perhaps, not surprising that FOG in the home might more closely resemble FOG during the simulated IADL tasks than FOG during the clinic FOG tasks. The clinic FOG tasks were designed to maximize the chance to elicit FOG. Some participants may have experienced FOG during these tasks but not during typical household activities. It is also not particularly surprising that Sensor-Based %ATSF in the home correlates more strongly with sensor-based measures from the lab than with video-based measures from the lab. Some differences are expected between video review and sensor measures when assessed at the same time [15]. Likewise, some differences are expected between sensor measures when taken at two different time points, as FOG severity may vary throughout the day. By comparing sensor-based measures in the home and video-based measures from the in-person visit, we are compounding these differences, and any correlation is therefore expected to be lower.

Wearable sensors are being used for a rapidly expanding range of health applications [29,30,31,32,33,34]. These measurement devices are particularly well-suited to gather specific information over long time periods in daily life. This approach is potentially fruitful to assess severity of freezing of gait because this symptom consists of stochastic events with high variability and context dependence and is thus potentially better assessed by collecting a large number of events compared to the few events that may occur during an office visit. Our results support the value of this approach.

The NFOG-Q showed very little correlation with objective measures of FOG throughout the study. This supports previous work showing that the NFOG-Q does not correlate with observed FOG in lab settings [13] nor at home monitoring of FOG [35]. Our sensor-based measurements, both in-person and at home, were much more closely related to the gold standard of expert video review during the in-person visit and the simulated-IADL task portion of the visit than the NFOG-Q. The differences seen between NFOG-Q and our sensor-based method may point to the important difference between patient perception and actual freezing severity. The NFOG-Q is measuring the patient’s perception of their freezing and is subject to recall bias, while the sensor-based method is using actual motion data to quantify freezing. The lack of correlation between these two measures may point to the variable patient perception of freezing, where some individuals perceive themselves to have worse freezing than the sensors detect while others perceive themselves to have less freezing severity than the sensors would suggest. Patient perception remains important, but this mismatch could allow for important discussions between patients and clinicians on how perceptions of freezing may be inaccurate.

Several limitations of this study should be noted. The sample size was relatively small (*n* = 19). With a larger sample of participants, we may have been able to detect more robust correlation patterns. The hardware used in this study was also not without limitations. The Physilog sensors’ ports became damaged after use by several participants. Both brands of sensors required overnight charging, as our detection algorithm required the devices’ highest sampling rate. Participants had to be thoroughly educated to ensure they knew how to charge the devices each night (see Appendixe B and Appendixe C). Devices with increased battery life and/or modifications to our approach to make it less energy intensive would allow for use of this method without overnight charging and would make it easier to implement reliably. We had to modify commercially purchased pouches to affix the sensors to the participants’ footwear. Products manufactured specifically for this purpose would also make our method easier to implement reliably. The detection algorithm does not yet include a user-friendly interface and required several hours of processing per participant. Despite these limitations, the correlations and accuracy values observed in this study are promising and suggest that our sensor-based FOG assessment method is feasible for use in the real world. Larger validation studies of our sensor-based method appear to be warranted, possibly with video recording in the home, to assess the accuracy of our method more confidently during real world use.

## 5. Conclusions

Our sensor-based FOG assessment method was able to produce high accuracy values during simulated real-world tasks when compared to the gold-standard video rater method. This high accuracy, along with correlation patterns between FOG assessment methods in lab and at home, suggests that our method may be useful for assessing FOG in the real world.

## Figures and Tables

**Table 1:** Participant Demographics, *n* = 19.

Participant Demographics, *n* = 19	
Age in years (mean ± SD)	71.95 ± 7.21
Sex (males, females)	M = 13, F = 6
MDS-UPDRS-III Score (mean ± SD)	41.89 ± 13.45
Hoehn and Yahr Stages (Stage 2, 3, 4)	*n* = 8, *n* = 9, *n* = 2
Levodopa Equivalent Daily Dose (mean ± SD)	955.2 ± 602.5
New Freezing of Gait Questionnaire Score	18.63 ± 4.98

**Table 2:** Sensor-Based FOG Detection Accuracy, *n* = 19.

Sensor-Based FOG Detection Accuracy, *n* = 19	
Vacuum Task	95.37% ± 5.03%
Dish Task	93.43% ± 10.48%
Sitting Task	98.60% ± 5.49%
Community Task	95.95% ± 5.29%
In-Person Lab Visit (Clinic FOG Tasks + Simulated IADL Tasks)	90.38% ± 8.57%

**Table 3:** Percent of Active Time Spent Freezing (%ATSF), *n* = 19.

Percent of Active Time Spent Freezing (%ATSF), *n* = 19	
Sensors: In-Person Visit (Clinic FOG Tasks + Simulated IADL Tasks)	6.36 ± 7.31
Sensors: Simulated IADL Tasks	1.84 ± 3.25
Sensors: Home	1.94 ± 3.06
Video Review: In-Person Visit (Clinic FOG Tasks + Simulated IADL Tasks)	11.22 ± 11.08
Video Review: Simulated IADL Tasks	1.90 ± 2.68

## Data Availability

Data is available upon request to the corresponding author subject to IRB restrictions and approval.

## Appendix A. Task Instructions

Instructions to Participant before beginning:

“We are going to ask you to do some walking, some turning, and a few chores and activities like those you might do at home. This is to determine whether you experience a phenomenon called ‘freezing of gait’ during these tasks. I will be reading instructions from a script. I will not be telling you how you’re doing. This is not a test of how well you move or how fast you walk. You don’t need to rush. For everything we ask you to do, we want you to do it at a comfortable pace. One of us will be near you at all times in case you need assistance with your balance. My instructions may sound robotic at times during these tasks. This is because we want to keep the instructions consistent for everyone. If you become dizzy or tired at any point and feel like you need to sit, let me know and I will pause the test and get a chair for you. Do you have any questions before we begin? [answer questions and ensure understanding.] Great.”

Notes for rater:

- For the dual task portion of each task, give the participant a number greater than 100.
- Read the directions slowly and clearly, giving plenty of time for participants to comprehend what is being said.

**Go Outside and Turn:**

Notes for rater: Start this task in the clinical exam room. Read instructions with participant sitting in chair facing door. Participants will start walking with toes at a marker 150 cm from door. If participant turns the wrong direction, do not tell them they made a mistake. Rather, for the subsequent trial, instead of simply saying “right” or “left,” say “to the right this time” or “to the left this time.” At end of each trial, once participant walks back into room (approximately halfway between door and starting position), say “stop.”

Instructions to participant:

[For Round 2, state “I will review the instructions for this task with you before we start it again.”]

1. “You can have a seat while I explain this task. When I say ‘ready, set, go,’ walk out the door into the open box marked on the floor in the middle of the hallway. You will turn around inside the box, walk out of the box, and walk all the way around the outside of the box, and then return to the room. When you walk through the door, I will tell you which direction to turn.
  - If I say to turn left, you will turn around inside the box toward your left like this [demonstrate]. You will then walk out of the box and make another left turn to walk around the outside of the box like this [demonstrate] before returning to the room.
  - If I say to turn right, you will turn around inside the box toward your right like this [demonstrate]. You will then walk out of the box and make another right turn to walk around the outside of the box like this [demonstrate] before returning to the room.
  Walk at a comfortable pace. Would you like me to demonstrate again? [Demonstrate if participant requests another demonstration.] Great, I will show you where to start. [Direct participant to assume starting position.]. Do you understand the instructions? [answer questions and ensure understanding before starting]. Great. When I want you to start, I will say “Ready, Set, Go.” Please wait for me to give the cue.”
2. “Please return to the starting position. [Direct participant.] I will tell you which direction to turn when you get to the door.”
3. Repeat above.
4. Repeat above.

-Sit and rest for at least 30 s-

5 “I will tell you which direction to turn when you get to the door. This time, I want you to count backward while you walk. I will give you a number to start with. You will count backward by three. Please count out loud. If you make a mistake with counting, continue counting down by three as if nothing happened. When I want you to start, I will say ‘ready, set, [and then your number.]’ So, for example, I might say ‘ready, set, 243.’ When I do this, you will start counting backward by three from the number I give you and start walking, like this: “243, 240, 237, and so on” [Demonstrate walking while counting down]. Walk at a comfortable pace. Do you have any questions? [answer questions and ensure understanding before starting] Great, please wait for me to give the cue.”
6 “Please return to the starting position. [Direct participant]. Start counting backward and walking when I give the cue.”
7 Repeat above.
8 Repeat above.

**Hallway Walk:**

Notes for rater: Start this task in the hallway. Read instructions with participant sitting in chair. Participants will walk to marker 6 m away from start, back to marker at 3 m away from start, and then return to marker 6 m away from start. Say “right and stop” or “left and stop” at end of task. If participant turns the wrong direction during a trial, do not tell them they made a mistake. Rather, wait until the end of that trial and then clarify instructions before beginning next trial. At the end of the trial, say “right and stop” or “left and stop.”

Instructions to participant:

[For Round 2, state “I will review the instructions for this task with you before we start it again.”]

1. “You can have a seat while I explain this task. When I say ‘ready, set, go,’ you will walk down the hallway. At some point while you’re walking, I will say “left” or “right.” [Begin walking away from participant.] When I say “left,” you will turn around toward your left like this [demonstrate turn] and continue walking in the opposite direction. [Begin walking away from participant.] When I say “right,” you will turn around toward your right like this [demonstrate] and continue walking in the opposite direction. Do not stop walking until I say the word “stop.” Walk at a comfortable pace. Would you like me to demonstrate again? [Demonstrate if participant requests another demonstration.] Great, I will show you where to start. [Direct participant to starting position.] Do you understand the instructions? [answer questions and ensure understanding before starting]. Great, please wait for me to give the cue.”
2. “We will start from here this time. [Direct participant.] Great, please wait for me to give the cue.”
3. Repeat above.
4. Repeat above.

-Sit and rest for at least 30 s-

5 “This time, I want you to count backward while you walk. I will give you a number to start with. You will count backward by three. Please count out loud. If you make a mistake with counting, continue counting down by three as if nothing happened. When I want you to start, I will say ‘ready, set, [and then your number.]’ So, for example, I might say ‘ready, set, 243.’ When I do this, you will start counting backward by three from the number I give you and start walking, like this: “243, 240, 237, and so on” [Demonstrate walking while counting down]. Walk at a comfortable pace. Do you have any questions? [answer questions and ensure understanding before starting.] Great, please wait for me to give the cue.
6 “We will start from here this time. [Direct participant]. Start counting backward and walking when I give the cue.”
7 Repeat above.
8 Repeat above.

**360 Degree Turns**:

Notes for rater: Start this task in the clinical exam room. Read instructions with participant sitting in chair. Participant will start task facing door with backs of heels on the 150 cm from door mark. If participant turns the wrong direction during a trial, do not tell them they made a mistake. Rather wait until the end of that trial and then clarify instructions before beginning next trial.

Instructions to participant:

[For Round 2, state “I will review the instructions for this task with you before we start it again.”]

1. “You can have a seat while I explain this task. When I say ‘right,’ I want you to make a complete turn to the right, like this [demonstrate]. When I say ‘left,’ I want you to make a complete turn to the left, like this [demonstrate]. In other words, each time I say ‘right’ or ‘left’ you will make a complete turn in that direction. Turn at a comfortable pace. If you start to feel dizzy, let me know, and we can pause the test. Would you like me to demonstrate again? [Demonstrate if participant requests another demonstration.] Great, I will show you where to start. [Direct participant to starting position.] Do you understand the instructions? [answer questions and ensure understanding before starting]. Great, please wait for me to give the cue.” [Cue: “Ready, set, right/left.”]
2. “Right/left”
3. “Right/left”
4. “Right/left” [After participant completes turn: “Ok you can stop and take a step back now.”]

-Sit and rest for at least 30 s-

5 “This time, I want you to count backward while you turn. I will give you a number to start with. You will count backward by three. Please count out loud. If you make a mistake with counting, continue counting down by three as if nothing happened. When I want you to start counting, I will say ‘ready, set, [and then your number.]’ So, for example, I might say ‘ready, set, 243.’ When I do this, you will start counting backward by three from the number I give you, like this: “243, 240, 237, and so on”. While you are counting backward, I will say either ‘right’ or ‘left’ several times and you will make a complete turn in whichever direction I say. Turn at a comfortable pace. Do you have any questions? [answer questions and ensure understanding before starting.] Great, please wait for me to give your number. [Cue: “Ready, set, number.”] [After participant successfully subtracts by three twice, state “right/left.”]
6 “Right/left”
7 “Right/left”
8 “Right/left” [After participant completes turn: “Ok you can stop and take a seat now.”]

**Activities of Daily Living** (**ADL**) **Tasks**

Notes for rater: Take hard chair into simulated apartment room. Participant will sit in hard chair while rater reads the following instructions and the instructions for the first ADL task. After reading instructions for first ADL task, remove hard chair from room, and place it outside in the hallway. The hard chair can be brought back into the room at any point if participant wants to sit.

Instructions to participant: “We will go over to the room next door for the next few tasks.” [Direct participant to room.] “You can have a seat here.” [Direct participant to chair.] For these next few tasks, we will be doing activities and chores which you might do at home or in the community to see whether you experience freezing of gait. Again, this is not a test of how well you move or how fast you walk. You don’t need to rush. If you get tired and need to sit at any point, let me know, and I will get the chair. Do you have any questions before we move on to the next task? [answer questions and ensure understanding.] Great.”

**Vacuum Task**:

Notes for rater: Start this task in the simulated apartment room. Place footstool far enough away from comfortable chair for participant to be able to vacuum between footstool and chair.

Instructions to participant: “For the next task, you will plug the vacuum in here. [Indicate the appropriate electrical outlet.] You will then vacuum this room. Try to vacuum every part of the room you can get to without moving furniture. Do not move any furniture for this task. When you finish, unplug the vacuum, and wrap the cord back up on the vacuum like it is now. The power button is here [indicate] and the foot release pedal is here [indicate]. Do you understand the instructions? [answer questions and ensure understanding before starting]. Great, you can begin.”

**Dish Task**:

Notes for rater: Place 4 plates, 4 bowls, 4 cups, 2 forks, 2 spoons, and 2 butter knives in the dishwasher, and then close the dishwasher before reading instructions. Have the cabinet spaces for each type of dish/silverware labelled (e.g., “plates here”) before beginning. Push footstool up against comfortable chair for this task. Start this task in the simulated apartment room.

Instructions to participant: “For the next task, you will unload the dishwasher and put the dishes away. Plates go over here and should be stacked on top of each other [indicate location.] Bowls go over here and should be stacked on top of each other on this shelf [indicate location.] Cups go over here on this shelf [indicate location.] Forks go here [indicate], spoons go here [indicate], and knives go here [indicate]. Only carry one dish or piece of silverware at a time. For example, don’t carry two forks at a time or two bowls at a time. Close the dishwasher when you finish. Do you understand the instructions? [answer questions and ensure understanding before starting]. Great, you can begin.”

**Community Task**:

Notes for rater: Start this task in the simulated apartment room.

Instructions to participant: “For the next task, you will walk to the elevator, take the elevator up to the first floor, get some hand sanitizer from the dispenser on the first floor, take the elevator back down to the basement, and then walk back to this room. I will walk with you. I won’t be giving you directions unless you ask me which way to turn. If you get lost and do not know which way to turn, you can ask me. Do you understand the instructions? [answer questions and ensure understanding before starting]. Great, you can begin.”

**Sitting Task**:

Notes for rater: Place 5 magazines and a bottle of hand sanitizer on small table or appliance near comfortable chair and set footrest in front of comfortable chair (approximately one foot from chair) before reading instructions. Start this task in the simulated apartment room.

Instructions to participant: “For this next part of the study, you will make yourself comfortable! You will sit in the rocking chair here and put your feet up on the footrest. Try to sit in whatever way is comfortable to you, like how you might sit at home. You can adjust the position of the footrest if you want, or you can ask us to adjust it for you. We want you to sit here for 10 min and read. Look through these magazines and find something to read for a few minutes. We will let you know when the 10 min have passed. Please use this hand sanitizer on your hands before and after touching the magazines. Do you understand the instructions? [answer questions and ensure understanding before starting]. Great, you can take a seat and begin.”

## Appendix B. Gait Up Physilog Home Instructions

        INSTRUCTIONS FOR SENSORS

For any questions, call at any time:

Pietro Mazzoni (phone number redacted) or David May (phone number redacted)

When you leave the lab today, you will be wearing 3 sensors: two clipped to your shoe laces, and one on your waist (left hip). Your sensor locations are:

  LEFT FOOT: _________   RIGHT FOOT: _______  WAIST: __________

Please wear these sensors for the rest of today and each of the next 3 days. Please wear your sneakers with the sensors for as much of the day as you can, starting after you get up in the morning and until you go to bed at night.

You can wear your sensors inside and outside the house, even if it’s raining. The sensors will not be damaged by a few splashes of water. However, do not submerge the sensors in water.

Please wear the sensors for the following days:

- The rest of the day after you leave the lab (Day 0: _________)
- The first day after your lab testing day (Day 1: _________)
- The second day after your lab testing day (Day 2: _________)
- The third day after your lab testing day (Day 3: _________)

**IN THE EVENING**:

- TAKE THE SENSORS OFF.
  - Take the hip sensor pouch off your belt or pants. Leave the shoe sensor pouches on the shoes. Take the sensors out of the pouches to charge them.
- TURN THE SENSORS OFF
  - Press the button on one sensor (any sensor) and hold it pressed until the light changes from green to orange (about 1 s). If you hold the button down too long (over 5 s) this will cause the sensors to reset, and any data you’ve collected will be lost.
  - Confirm the light is steadily off. If the light is flashing, repeat the above step or call Pietro or David (numbers above)
  - Repeat the above steps for the remaining 2 sensors
- CHARGE THE SENSORS
  - Plug a cable into each sensor and connect to a wall outlet
  - The sensors will flash purple for a few hours. They will change to steady green to indicate they have completed charging.

**IN THE MORNING**:

- CONFIRM THE SENSORS ARE FULLY CHARGED.
  - If they are fully charged, the light on each sensor will be a steady green, and you can unplug the sensors.
  - If the lights are not steady green, let them keep charging and call Pietro or David (numbers above).
- TURN THE SENSORS ON
  - Press the button on one sensor (any sensor) and hold it pressed until the green light turns off (about 1 s)
  - Confirm that the light is flashing green (about every second; each flash is very fast and faint; this is normal).
  - After a few seconds, the 3 sensors’ lights should be flashing green all at the same time.
  - If the 3 lights are not flashing green at the same time, turn each sensor off and repeat the above steps, or call Pietro Mazzoni (number above)
- WEAR THE SENSORS
  - Fasten the hip sensor pouch on your belt, near your left hip
  - Place each sensor in its pouch.
    ○ Be sure to put the left foot sensor **on the participant’s left foot** and the right foot sensor **on the participant’s right foot**.
    ○ Be sure to put each sensor in its pouch with the sticker facing away from the participant’s body. Do not put the sensors in the pouch upside down. See images below.

**AT THE END OF DAY 3**:

- At the end of day 3: turn the sensors off and charge them. Let them stay plugged in and charging until a research team member is ready to pick them up.
- Take the pouches off your shoes and pants or belt. If you cannot get the pouches off the shoes, a research team member will be happy to do it for you when we pick the sensors up.
- Place the 3 sensors, the pouches, and the charging cables in the box provided.
- Wait for a phone call from Pietro or David who will provide instructions on how to return the sensors

       Thank you for participating in our study!

## Appendix C. Actigraph Link Home Instructions

        INSTRUCTIONS FOR SENSORS

For any questions, call at any time:

Pietro Mazzoni (phone number redacted) or David May (phone number redacted)

When you leave the lab today, you will be wearing 3 sensors: two clipped to your shoe laces, and one on your waist (left hip). Your sensor locations are:

  LEFT FOOT: _________   RIGHT FOOT: _______  WAIST: __________

Please wear these sensors for the rest of today and each of the next 3 days. Please wear your sneakers with the sensors for as much of the day as you can, starting after you get up in the morning and until you go to bed at night.

You can wear your sensors inside and outside the house, even if it’s raining. The sensors will not be damaged by a few splashes of water. However, do not submerge the sensors in water.

Please wear the sensors for the following days:

- The rest of the day after you leave the lab (Day 0: _________)
- The first day after your lab testing day (Day 1: _________)
- The second day after your lab testing day (Day 2: _________)
- The third day after your lab testing day (Day 3: _________)

**SENSOR ORIENTATION**

This is the front side (screen side) of the sensors.

This is the back side (tape side) of the sensors.

Each sensor is labelled on the back with tape to show you where to place them on the study participant.

- The left foot sensor goes on the participant’s left foot.
- The right foot sensor goes on the participant’s right foot.
- The hip sensor goes on the participant’s left hip (clipped to belt).

**IN THE EVENING**:

- TAKE THE SENSORS OFF.
  - Take the hip sensor pouch off your belt or pants. Leave the shoe sensor pouches on the shoes. Take the sensors out of the pouches to charge them.
- CHARGE THE SENSORS
  - Plug each of the three charging docks in to a wall outlet
  - Place each sensor on a charging dock as shown below.
  - Charging dock should light up orange. This will change to green when they are fully charged.

When charging, back of sensor (tape) should face toward charging cable.

**IN THE MORNING**:

- CONFIRM THE SENSORS ARE FULLY CHARGED.
  - If they are fully charged, the light on each charging dock will be a steady green, and you can unplug the sensors.
  - If the lights are not steady green, let them keep charging and call Pietro or David (numbers above).
- WEAR THE SENSORS
  - Fasten the hip sensor pouch on your belt, near your left hip
  Place each sensor in its pouch.
    ○ Be sure to put the left foot sensor **on the participant’s left foot** and the right foot sensor **on the participant’s right foot**.
    ○ Be sure to put each sensor in its pouch with the front side (screen side) facing away from the participant’s body. Do not put the sensors in the pouch upside down. See images below.

**AT THE END OF DAY 3**:

- At the end of day 3: place the sensors on the chargers. Let them stay plugged in and charging until a research team member is ready to pick them up.
- A research team member will assist you with taking the pouches off the shoes unless otherwise instructed.
- Place the 3 sensors, the pouches, the charging docks, and charging cables in the box provided.
- Wait for a phone call from Pietro or David who will provide instructions on how to return the sensors.
